# Supplementary material for: Predicting the Potential Global Distribution of the Plum Fruit Moth Grapholita funebrana Treitscheke Using Ensemble Models
Source: Insects. 2024 Aug 30;15(9):663. doi: 10.3390/insects15090663 (PMC11432621; doi:10.3390/insects15090663)
Supplement: Supplementary file 1 [file insects-15-00663-s001.zip › supplementary files/Table S3 The environmental variables considered in this study.pdf]

Table S3 The environmental variables considered in this study

**Variable type**	**Variable**	**Description**
Bioclimatic Variables	**BIO01**	Annual mean temperature
	BIO02	Mean diurnal range (mean of monthly (max temp-min temp))
	**BIO03**	Isothermality (bio2/bio7) (× 100)
	**BIO04**	Temperature seasonality (standard deviation ×100)
	**BIO05**	Max temperature of warmest month
	**BIO06**	Min temperature of coldest month
	**BIO07**	Annual temperature range (bio5–bio6)
	BIO08	Mean temperature of wettest quarter
	BIO09	Mean temperature of driest quarter
	**BIO10**	Mean temperature of warmest quarter
	**BIO11**	Mean temperature of coldest quarter
	BIO12	Annual precipitation
	BIO13	Precipitation of wettest month
	BIO14	Precipitation of driest month
	BIO15	Precipitation seasonality (coefficient of variation)
	BIO16	Precipitation of wettest quarter
	BIO17	Precipitation of driest quarter
	BIO18	Precipitation of warmest quarter
	BIO19	Precipitation of coldest quarter
Elevation	**elev**	Ground height above sea level (m)

Note: the variables in bold were finally used in the modelling.
