# Supplementary figures and images for: Predicting the Potential Global Distribution of the Plum Fruit Moth Grapholita funebrana Treitscheke Using Ensemble Models
Source: Insects. 2024 Aug 30;15(9):663. doi: 10.3390/insects15090663 (PMC11432621; doi:10.3390/insects15090663)

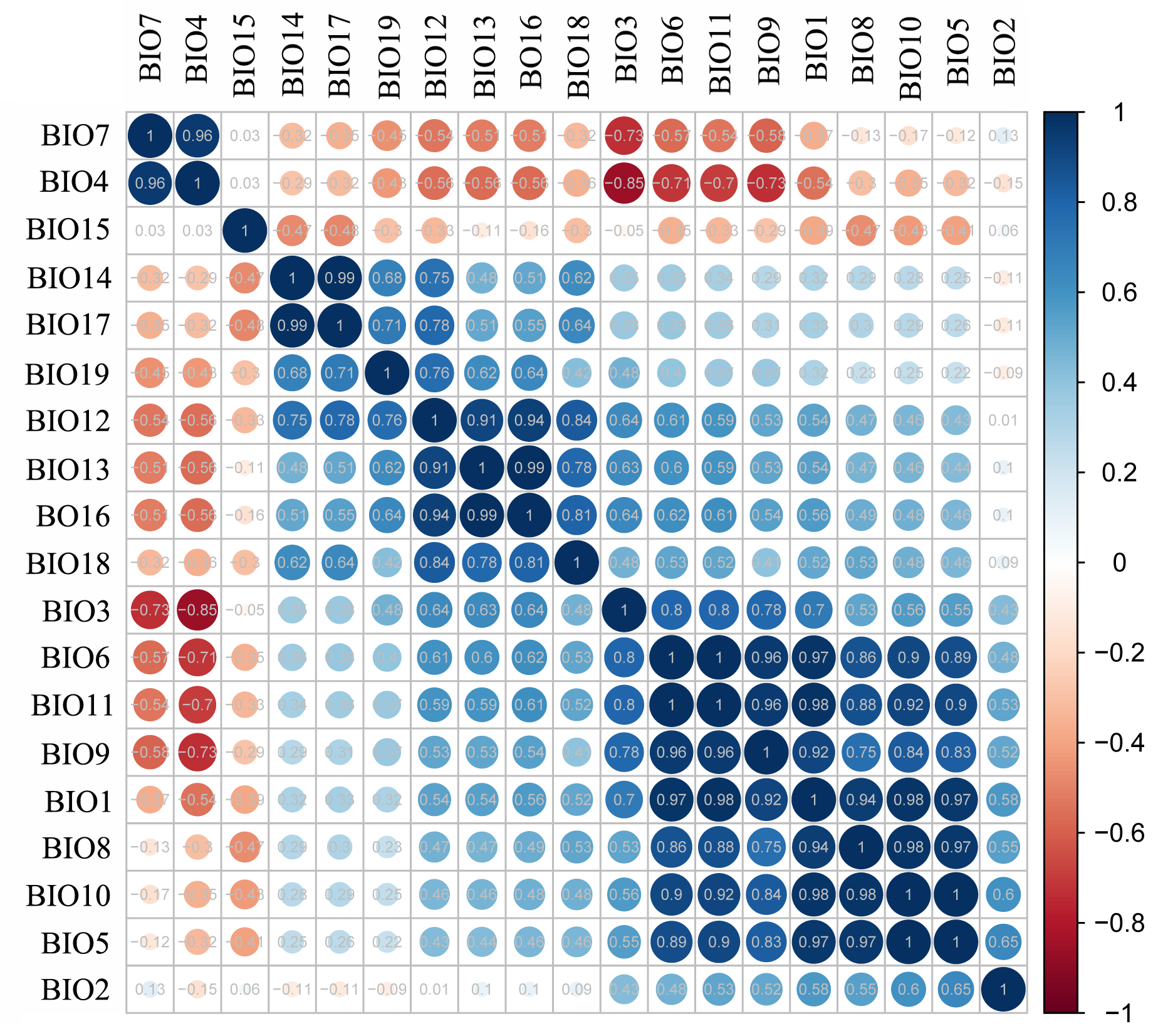

Supplement: Supplementary file 1 [file insects-15-00663-s001.zip › supplementary files/Figure S1 The Pearson’s correlation analyses of 19 bioclimatic variables.tif]

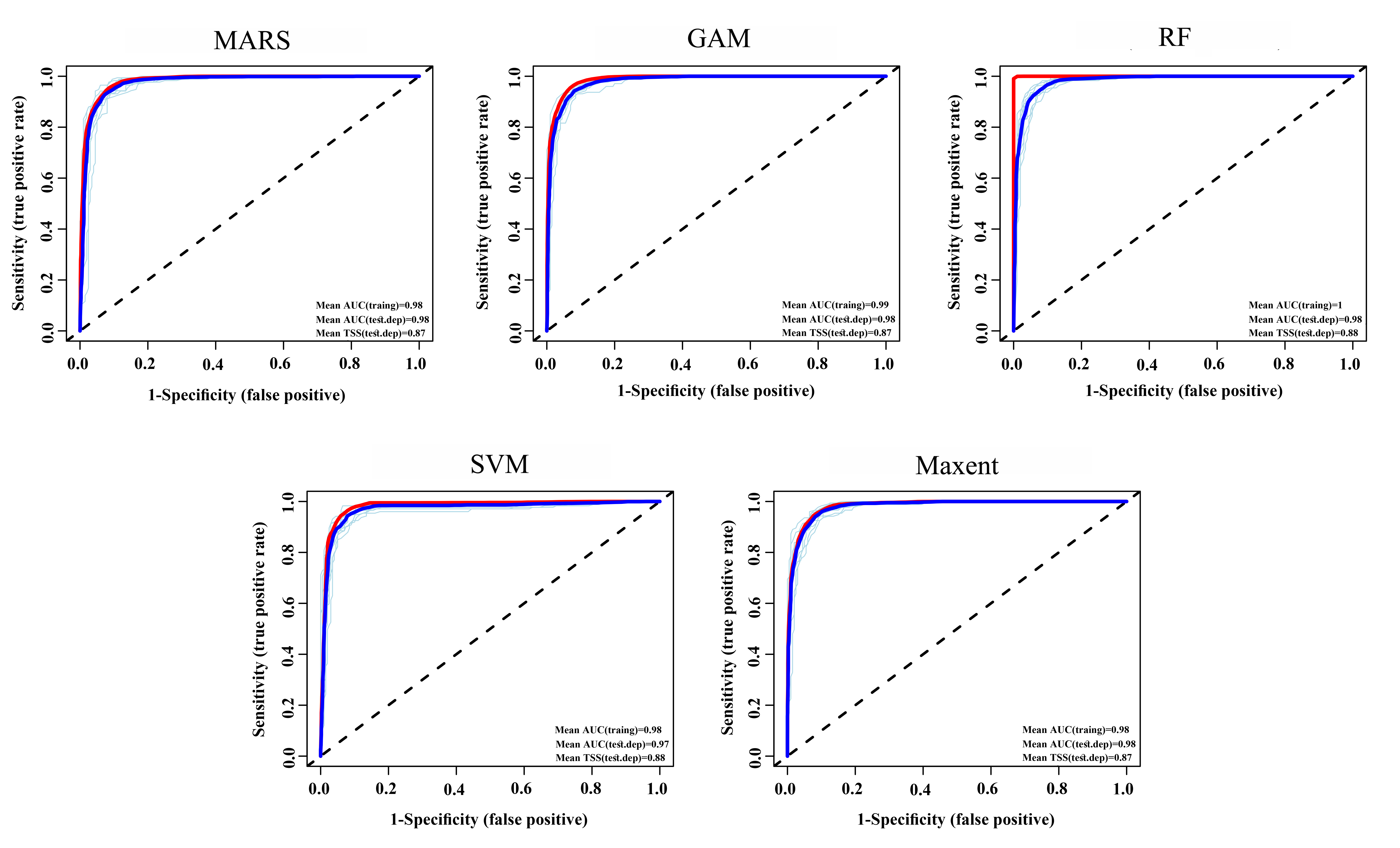

Supplement: Supplementary file 1 [file insects-15-00663-s001.zip › supplementary files/Figure S2 The area under the receiver operating characteristic curve (AUC) and true skill statistics (TSS) values for five used models under BIOs+elev .tif]
